# Breaking the Cycle: Understanding Frequent Relapses in Alcohol Treatment – The Impact of Patient Engagement and Prevention Strategies through Retrospective Analysis

**DOI:** 10.1192/j.eurpsy.2025.937

**Published:** 2025-08-26

**Authors:** M. Cameira, Â. Ferreira, I. Pereira, M. Andrade, S. Morais, J. Teixeira

**Affiliations:** 1 ULS São José - Hospital Júlio de Matos, Lisboa; 2 ULS Arrábida - Hospital de São Bernardo, Setúbal; 3 ULS Almada-Seixal- Hospital Garcia de Orta, Almada, Portugal

## Abstract

**Introduction:**

Alcohol use disorder is a significant Public Health issue with substantial socioeconomic impact, morbidity, and mortality. Achieving therapeutic success remains challenging due to frequent treatment dropouts, relapses, and readmissions. It is estimated that 20-40% of patients discontinue detoxification treatment prematurely, with some studies highlighting the influence of psychiatric comorbidities, polysubstance use, unemployment, and impulsivity. Premature discontinuation increases the risk of medical complications and worsens prognosis. Although anti-craving medications have demonstrated efficacy in preventing relapse, they remain underprescribed.

**Objectives:**

This study aims to identify and analyze determinants of readmissions, particularly in patients with a history of treatment dropout, and propose strategies to enhance therapeutic success.

**Methods:**

A two-year retrospective analysis was conducted on readmitted patients, with a focus on previous dropouts, within the 12 months prior to their last hospitalization for alcohol detoxification at ULS São José - Hospital Júlio de Matos. The analysis included sociodemographic data, comorbidities, anti-craving therapy, integration into therapeutic communities (TC), and post-discharge outcomes.

**Results:**

A total of 37 patients were identified with readmissions in the past 12 months. Of these, 9 (24.3%) had left their previous admission against medical advice, most of whom had experienced 4 or more admissions. There was a similar gender distribution, with all patients being single or divorced, 66.7% unemployed, and the same percentage experiencing economic hardship. Two-thirds had psychiatric comorbidities. None had recently received anti-craving medication, and only 2 (22.2%) had prior involvement with a TC. During their readmission, 44% were discharged to a TC or Day Center, and 2 left again against medical advice.

**Conclusions:**

It was found that many frequent users discharged themselves against medical advice from previous hospitalizations. These patients showed a high prevalence of socioeconomic problems and psychiatric comorbidities, with none receiving anti-craving therapy. In the future, it will be essential to analyze the reasons for these dropouts to improve the effectiveness of treatment during hospitalization. It is concluded that therapeutic plans must be personalized and tailored to the patients’ multiple needs to ensure better adherence.

**Disclosure of Interest:**

None Declared

